# Automatic and Efficient Fall Risk Assessment Based on Machine Learning

**DOI:** 10.3390/s22041557

**Published:** 2022-02-17

**Authors:** Nadav Eichler, Shmuel Raz, Adi Toledano-Shubi, Daphna Livne, Ilan Shimshoni, Hagit Hel-Or

**Affiliations:** 1 Department of Computer Science, University of Haifa, Haifa 3498838, Israel; hagit@cs.haifa.ac.il; 2 Department of Information Systems, University of Haifa, Haifa 3498838, Israel; razshmu@gmail.com (S.R.); ishimshoni@is.haifa.ac.il (I.S.); 3 Physiotherapy Institute, Galilee Medical Center, Chicago, IL 60639, USA; adit@gmc.gov.il (A.T.-S.); daphnaniv@gmail.com (D.L.)

**Keywords:** fall risk detection, balance, Berg Balance Scale, human tracking, elderly, telemedicine, diagnosis

## Abstract

Automating fall risk assessment, in an efficient, non-invasive manner, specifically in the elderly population, serves as an efficient means for implementing wide screening of individuals for fall risk and determining their need for participation in fall prevention programs. We present an automated and efficient system for fall risk assessment based on a multi-depth camera human motion tracking system, which captures patients performing the well-known and validated Berg Balance Scale (BBS). Trained machine learning classifiers predict the patient’s 14 scores of the BBS by extracting spatio-temporal features from the captured human motion records. Additionally, we used machine learning tools to develop fall risk predictors that enable reducing the number of BBS tasks required to assess fall risk, from 14 to 4–6 tasks, without compromising the quality and accuracy of the BBS assessment. The reduced battery, termed Efficient-BBS (E-BBS), can be performed by physiotherapists in a traditional setting or deployed using our automated system, allowing an efficient and effective BBS evaluation. We report on a pilot study, run in a major hospital, including accuracy and statistical evaluations. We show the accuracy and confidence levels of the E-BBS, as well as the average number of BBS tasks required to reach the accuracy thresholds. The trained E-BBS system was shown to reduce the number of tasks in the BBS test by approximately 50% while maintaining 97% accuracy. The presented approach enables a wide screening of individuals for fall risk in a manner that does not require significant time or resources from the medical community. Furthermore, the technology and machine learning algorithms can be implemented on other batteries of medical tests and evaluations.

## 1. Introduction

Accidental falls are a major concern in the elderly population, often requiring hospitalization, and may lead to death [1,2]. Falls are one of the main causes of disability, loss of independence, and reduced quality of life. This incurs high expenses on the individuals, their families, and the public health system [3,4]. It has been shown, however, that individuals can significantly reduce the risk of fall by participating in fall prevention programs [5,6]. Thus, there is great importance in performing a wide screening of the elderly population for the risk of fall and, consequently, initiating appropriate intervention programs.

Assessing the risk of fall is typically performed by physiotherapists and other types of medical professionals using various standardized and validated balance tests. One such test is the Berg Balance Scale (BBS) [7,8], a rigorous and time-consuming examination, since it requires the patient to perform 14 different tests. Due to its demand on the medical professional resources, these tests are not widely performed on the general public and are typically administered in the context of rehabilitation. Thus, more efficient testing methods for the risk of fall are crucial for implementing community-wide screening to identify high-risk individuals [5,6].

In this paper, we present a method to alleviate the workload in fall risk assessment. We developed our methods for the Berg Balance Scale (BBS); however, the approach is applicable to any time-consuming battery of tests. We developed an automated system for assessing the risk of fall using the BBS test, which is non-invasive and easy to use. It uses a novel self-calibrating multi-depth camera human motion tracking system previously developed by the authors. Using the data extracted from the cameras, machine learning classifiers were developed to evaluate the performance of the tasks by the patient. Thus, a medical professional is no longer needed to monitor and assess the performance of the test by the patient.

Still, performing 14 tasks is time consuming. Thus, in this paper, we present a machine-learning-based method to predict the fall risk, which enables reducing the number of BBS tasks required to assess fall risk from 14 to 4–6 tasks while maintaining the quality and accuracy of the BBS assessment (at 96%). We term the reordered and reduced BBS battery Efficient-BBS (E-BBS), as it reduces the the number of tasks to be performed and consequently reduces the time required to complete the BBS test. We present the E-BBS task ordering methods, which proceed either in a predefined order of tasks or on a per-patient adaptive task sequencing. The E-BBS can be performed by physiotherapists in a traditional setting or deployed using the automated system, allowing an efficient and effective BBS evaluation.

The automated system was tested in a major hospital, under the guidelines of the Declaration of Helsinki. The results showed high accuracy rates in predicting fall risk and showed a correlation with the BBS scores on individual BBS motion tasks as assessed by medical professionals. The E-BBS was developed by training machine learning algorithms on the data collected at the hospital. The trained E-BBS system was shown to reduce the number of tasks in the BBS test by approximately 50% while maintaining 97% accuracy.

The main scientific contribution of the paper is the novel approach to shortening and creating an adaptive sequence of testing from any given battery of tests (medical or other). The paper implemented the approach on the BBS test, but it can be exploited to reduce any battery of tasks that provides a final score or outcome to a shorter test while maintaining accuracy. The outcome of the study will also hopefully contribute to the medical community, allowing more efficient testing of the risk of fall that can be deployed in medical centers, community centers, as well as in private homes. It will allow a wider reach to the aging community and, as such, help to improve this population’s welfare and quality of life together with reducing the burden on families, communities, and at the national level as well.

In the following sections, we review the automated system and introduce the E-BBS. We present a description of the full system including a review of the previously presented study in [9] with additional statistical analysis. We show the results of a pilot study, run on 130 patients in a major hospital, including the accuracy and statistical evaluations. We then present the E-BBS system and show its accuracy and its confidence levels, as well as the average number of BBS tasks that are required to reach the accuracy thresholds.

There is a plethora of balance and risk of fall tests that have been validated and are used in the medical community (see [10] for a review). Most tests involve motor tasks that are scored by a physiotherapist or medical professional. The motor tasks are mostly related to daily actions and movements that are typically performed by humans such as walking, rising from a chair, transitioning between sitting and standing positions, reaching, and more. Some tests are short and easy to administer; others are longer and include a battery of tasks, but are more comprehensive and systematic.

Short tests that focus on walking assess the time or distance required to complete the task and include the 2 m walk [11], 10 m walk [12], and 6 min walk [13]. A more comprehensive gait test is the Dynamic Gait Index [14] with several motor tasks of increasing difficulty.

Tests relying on transitioning into and out of a chair are also very common as this action is important in daily life. They assess lower body strength [15], which is related to the risk of fall [16]. The single task tests in this class include the 30 s chair stand [15], 5X-Sit-to-Stand [17], and 10X-Sit-to-Stand, requiring subjects to rise and lower themselves into a chair as fast as possible. The number of repetitions performed or the time to perform a set number of repetitions serves as the score in these tests.

A very popular balance test, combining both walking and transitioning from a chair, is the Timed Up and Go Test (TuG) [18,19]. It measures the time required to rise from a chair, walk 3 m, turn, return to the chair, and sit back down [20]. This test is popular as it is short and easy to administer, though for reliability, it is often repeated several times [21].

Another type of balance test is those based on static pose including the Unipedal Stance Test [22], Unilateral Forefoot Balance Test [23], and the Romberg Test [24]. These tests have subjects stand on one or both feet in different positions (aligned, tandem, or toe to heel) and with eyes opened or closed. Combining several of these poses in increasing difficulty is used in the 4-Stage Balance Test [25].

Finally, balance tests based on in-place stepping include the Step Test [26], where one foot is repeatedly placed on and off a step, the Four Square Step Test [27,28], where a sequence of steps is performed over objects in a square path, and the Y Balance Test [29], where subjects perform lunging steps in three direction.

The above-described tests rely on a single task or on very few tasks. Though requiring little time to administer, they are not, in general, comprehensive and rigorous. For diagnosis and referral to treatment, medical professionals typically use a more comprehensive testing scheme that includes a larger number of tasks. Though more informative, these tasks are, unfortunately, more time consuming. Common balance tests in this class include the Berg Balance Scale (BBS) [7], the Tinetti Assessment Tool (TAT) [30], the Short Physical Performance Battery (SPPB) [31], and the Balance Evaluation Systems Test (BESTest) [32]. These tests each include a battery of tasks involving holding a pose, walking, sit-to-stand transitions, and more.

As a compromise between comprehensive testing and test administration time, two approaches have been taken. For several of the lengthy tests, shorter versions have been introduced and validated such as the MiniBest [33] and the Short-BBS (SFBBS) [34] (see below). The second approach attempts to incorporate technology and advanced algorithms to assist or replace the balance test. Various sensors have been used to track individuals in their natural environment and assess their balance and risk of fall. Examples include wearable sensors [35], inertial sensors [36], and visual sensors [37,38]. Unfortunately, these intrusive methods are often uncomfortable and expensive and typically do not provide a comprehensive analysis of the patient’s balance (e.g., type of imbalance and physiological source of the imbalance). Cameras and other non-contact sensors are advantageous, in being non-intrusive and being capable of collecting a wide range of data per patient. These non-intrusive sensors are desirable for hospitals, old age homes, and home care systems [39,40]. However, video cameras do not capture depth information, which, in assessing balance, may lead to erroneous outcomes and incorrect assessment of the risk of fall [41]. Depth-sensing cameras (such as the Microsoft Kinect [42] and others) can be used to capture depth in the scenes using technologies such as stereo imaging, structured light, and time-of-flight technologies [43]. Indeed, depth sensors have been used on single-task balance tests including the Get-Up-and-Go [44], 10-meter walk test [45], Single-Legged Stance Test [46], and on gait assessment [47]. However, many of the multi-task balance tests require pose and motions that give rise to self-occlusion (for example, the 360° turn in the BBS assessment), in which case multiple cameras are required. However, using more than a single camera requires calibration and synchronization [38], which is inappropriate for an easy-to-use balance assessment system. In our system, we used two depth-sensing cameras in a novel multi-depth camera tracking system, which performs synchronization and calibration automatically and requires no manual intervention [48]. Using this non-invasive technology together with Machine-Learning (ML)-based algorithms, balance and the risk of fall can be successfully and efficiently assessed.

### 1.1. The Berg Balance Scale

In this study, we implemented our approach on the Berg Balance Scale (BBS) [7,8], a standard and validated measure commonly used by medical professionals to assess the risk of fall.

The BBS is comprehensive and includes 14 motor tasks of varying difficulty, with tasks involving sitting and rising from a chair, holding a pose, turning, stepping, and more. Each task is scored on a five-level scale ranging from zero (unable) to four (independent). The final BBS score is obtained by summing the 14 individual task scores [8]. A BBS score of 36 or less implies a near 100% chance of fall within 6 mo [14]. Scores from 0–20 are considered high fall risk, from 21–40 medium fall risk and scores from 41–56 as low fall risk [7,8].

The BBS measure has been well studied. It has been shown to be valid and to have high sensitivity [14,33,49]. Test–retest reliability has been shown to be very good when tested on elderly individuals [50,51], stroke patients [52,53], and Parkinson’s patients [54,55]. The inter- and intra-rater reliability of the BBS was also shown to be good when tested on elderly individuals [7,8,33,56,57], Parkinson’s patients [55,58], stroke patients [59,60], and patients following spinal cord injury [61].

The BBS, though comprehensive, is time consuming. To compensate for the lengthy testing, a short form of the BBS was proposed (SFBBS) [34]. This test includes seven of the fourteen BBS tasks, and the rating is on a three-point scale (vs. the five-point scale of the BBS). The SFBBS was shown to have good validity, internal consistency, and reliability on stroke patients [34,62] and on the elderly [63,64] and has been shown to compare well with the standard BBS [62,64,65]. In this paper, we present the Efficient-BBS (E-BBS), an adaptive BBS testing scheme based on machine learning, and show that it significantly improves performance over the SFBBS.

## 2. Automated Fall Risk Assessment System

The BBS balance assessment task is highly time consuming and thus requires significant resources of the medical professional and of the medical organization as a whole. Currently, this test is most often administered to patients who have already undergone a fall or a medical procedure (stroke, hip/knee replacement, etc.) in order to assess the severity of their condition or assess their rehabilitation. Although it has been shown that timely intervention can reduce the risk of fall, detecting those individuals from the general population that are at risk and would benefit from this intervention is not easily possible, given the expense of balance assessment.

Thus, we propose to develop an automated fall risk assessment system, which is able to administer the BBS procedure and, using machine learning (ML) methods, to automatically predict the risk of fall of the subject. This can be performed without the intervention of a medical specialist and thus can be used for mass screening. Furthermore, since running the complete battery of 14 BBS tasks is time consuming, we propose a method for using a minimal number of BBS tasks that will maintain the accuracy of the standard BBS assessment while significantly reducing the test time.

**Figure 1:**
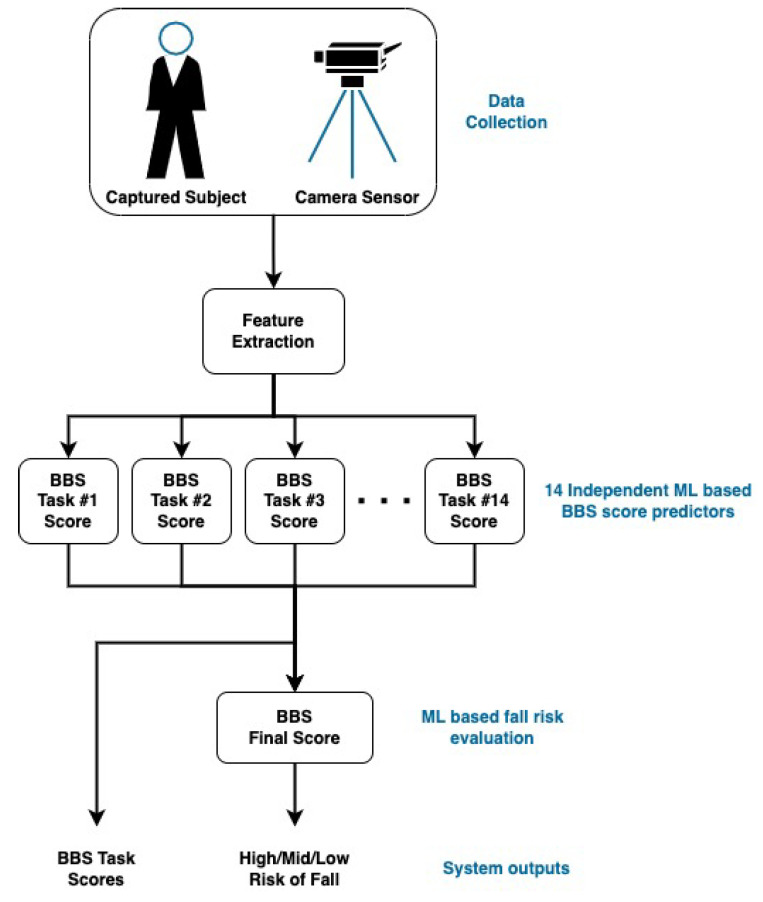
Schematic diagram of the BBS score and fall risk prediction system.

To be widely used, outside medical centers, the system must be non-intrusive, portable, and easy to use, while still maintaining reliable and consistent BBS score predictions. The proposed system consists of three major components (see Figure 1):

1. Motion tracking system, including 3D cameras;
2. Automatic BBS score prediction algorithms;
3. Final fall risk assessment using machine learning.

The first two components compute the 14 BBS scores by tracking the subject’s motion and using machine learning to predict the scores. This work, which was presented in [9], is reviewed in Section 3 and Section 4. Section 4 also reviews the machine learning model used to predict the level of risk from the 14 previously predicted BBS scores either as a final score (from 0–56) or as one of three levels of risk (high, medium, or low risk of fall). Finally, in Section 5, we describe our novel machine-learning-based approach for predicting the final BBS score, the E-BBS, which uses an adaptively chosen subset of BBS tasks per subject, based on the subject’s scores on these tasks. This approach reduces the number of tasks required to 4–6 tasks per subject.

## 3. Motion Capture and Tracking

**Figure 2:**
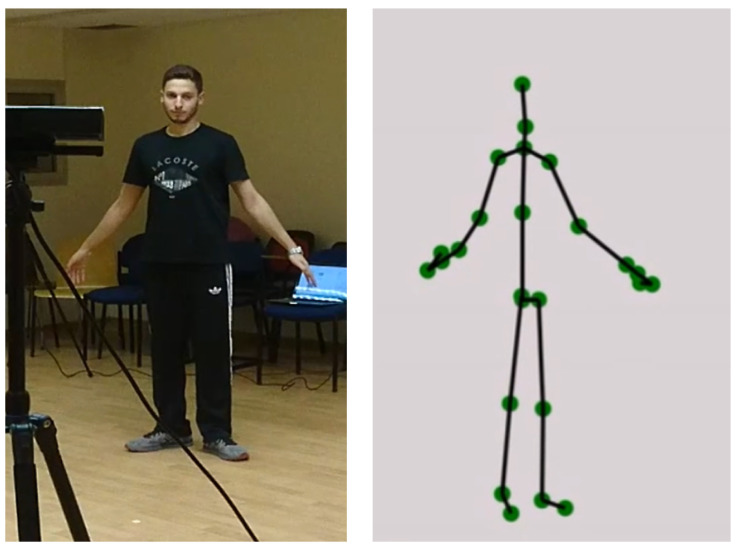
The 3D sensor (**left**) measures the distances of points in the scene from which a skeleton representation of the body pose is produced (**right**).

To track subjects performing the BBS tasks, we used the Microsoft Kinect [42], a depth sensor camera based on time-of-flight technology [66]. It provides depth information, i.e., the distance from the camera, for every point in the scene for each video frame. When filming human subjects, a skeletal body representation composed of 3D joints and connecting bones (Figure 2) is extracted from the captured depth information using machine learning algorithms [67,68,69]. For the purpose of tracking and estimating BBS task performance, we also collected the 3D data points in the patient’s immediate surroundings, floor position, and orientation, as well as the 3D points of objects in the scene relevant to the BBS task.

Due to the possibility of the self-occlusion of the body during some of the BBS tasks and to ensure full coverage of the subject, we used a two-camera setup where two cameras were placed 3 m from the subject, about 2 m apart and at 45° angles. This ensured full coverage, as well as merging of the data to reduce noise and uncertainty in the skeletal structure.

A major drawback of any multi-camera system is the necessity of performing synchronization and calibration between the cameras. This process typically requires a specialized calibration session with specific calibration tools, a process that is impractical and infeasible for systems such as ours that are targeted for use in the community.

Thus, we used a novel multi-camera tracking system developed by our team [48,70] in which synchronization and calibration are performed automatically and on the fly by exploiting the patient’s motion. The skeletal data acquired by the two calibrated cameras can then be easily integrated. Using this multi-depth-sensing camera tracking system allows motion and pose tracking of subjects to be non-intrusive, portable, and inexpensive.

Kinect allows motion and pose tracking of subjects to be non-intrusive, portable and inexpensive, motion capture system Motion tracking is thus performed non-intrusively.

## 4. Predicting BBS Scores Using Machine Learning

In this section, we review the system we developed based on computer vision tools and machine learning to predict the BBS scores of a patient on each of the 14 BBS tasks, as well as to predict the final risk of fall. The predicted scores were shown to correlate well with the scores assessed by the physiotherapists. More details can be found in [9].

Following the data collection, spatio-temporal features were extracted from the collected skeletal data and used to train a machine learning model to predict each of the 14 BBS task scores. Given the 14 predicted scores, an additional model was trained to predict the final risk of fall of the patients. Figure 1 shows a diagram of the automated system.

### 4.1. Data Collection

Data for this project were collected in the Physiotherapy Unit at a major public hospital under the guidelines of the Declaration of Helsinki (ID: 0194-15-NHR, Galilee Medical Center). A total of 130 subjects were recruited, 100 of whom were hospital in-patients. Thirty of the subjects were visitors or care givers of patients and were recruited as subjects of low fall risk. All subjects (in-patients and controls) were aged 65 or older. Seventy-six of the subjects were female, and fifty-three were male subjects. All subjects took the BBS test in the hospital’s physiotherapy room. The multi-camera tracking system (Section 3) recorded the subjects performing the 14 BBS tasks. Two physiotherapists administered and scored the patient on each of the 14 tasks. The double scoring by the physiotherapists’ served to validate the scores. Due to the high BBS inter-rater reliability [7,8,33], only seldomly were the scores of the two therapists inconsistent; in these cases, the more conservative score was used. The physiotherapists’ BBS scores for each patient served as the ground truth labels for training the learning models.

### 4.2. Feature Extraction

**Figure 3:**
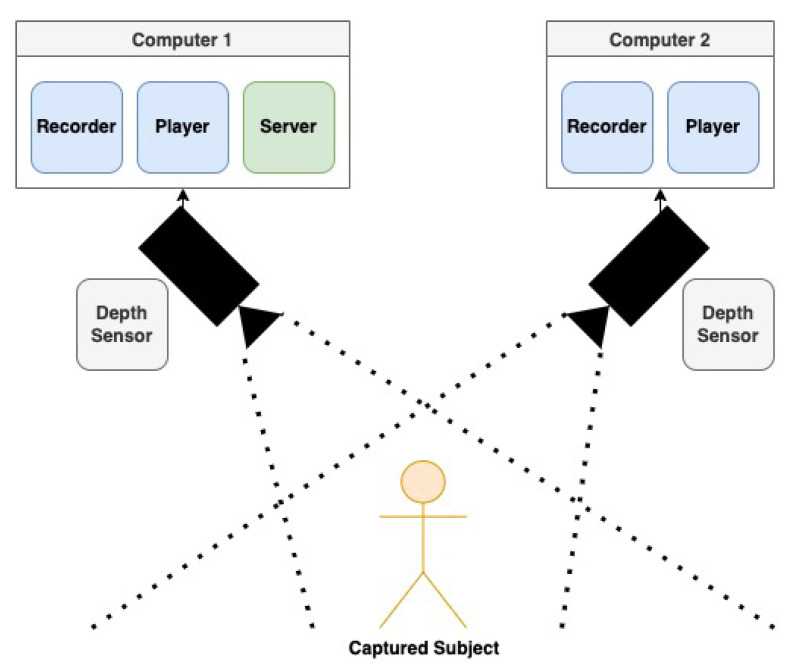
The multi-camera tracking system setup includes two depth sensors allowing the capture of the full range of patient motion, as well as enabling data merging to reduce noise and skeleton errors.

**Figure 4:**
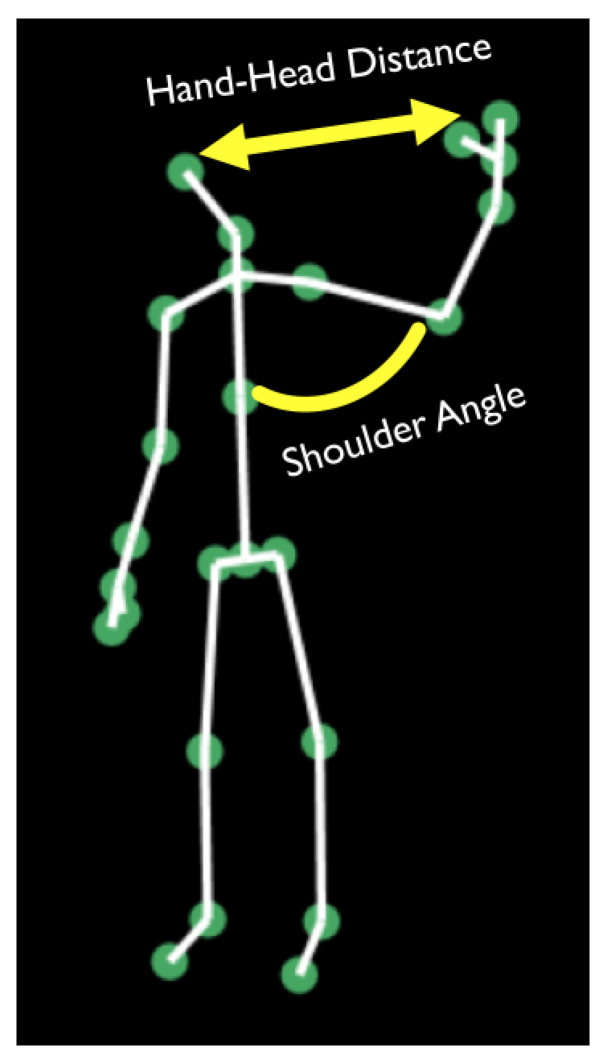
Spatio-temporal features are computed from the skeleton data in each recorded video frame.

To train the BBS score prediction models, sets of features were defined for each of the 14 BBS tasks. The skeletal sequence acquired for each subject per each BBS task (Figure 3) served as the basis for the features. Feature extraction was performed in two steps. First, features were extracted from the skeletal structure of each frame in the sequence. These included: relative positions of skeleton joints, angles between connecting bones, distances between body parts, heights of joints from the ground, and more (Figure 4). Most of the extracted features were independent of the location of the subject and invariant to body size.

In the second step, spatio-temporal features were calculated from these per-frame features including: maximal/minimal/mean values of the per-frame features across all frames in the sequence, average speed and acceleration of joints across the sequence, motion-paths of the joints, and more. This set of spatio-temporal features served to represent the motion action of a subject performing a single BBS task and were used to train the machine learning algorithms.

To improve model training, the number of features was reduced by selecting the most informative features per BBS task, as computationally derived from the trained models. Feature selection was also guided by recommendations from the physiotherapists as to the most predictive parts of the body and its features. Feature selection resulted in different features per each BBS task, ranging from 100–200 features (for examples, see [9,71]).

### 4.3. Training

Training and testing were performed using the data collected at the hospital of patients performing the 14 tasks of the BBS test. Each task was recorded as a skeletal sequence, represented using the features described above, and labeled with the BBS score assigned by the physiotherapists. Separate models were trained to predict the BBS score for each of the 14 BBS tasks. An additional model was trained on the BBS scores to predict the final BBS fall risk assessment.

For each of the 14 tasks, a random forest classifier [72,73] was trained using leave-one-out cross-validation [74]. The model hyper-parameters were sought using grid search [75]. The number of trees was set to 100 and the depth to 10. The random forest classifier was chosen as its use of bootstrapping enables these models to work well on small datasets. Furthermore, the random forest classifier allows feature ranking [76,77] in which the predictive power of features can be assessed. This in turn assists in feature selection to assist in further reducing over-fitting.

An additional ML-based classifier was trained on the 14 scores predicted by the random forests, to predict the final risk of fall (Figure 1). The risk of fall is defined as one of three categories based on the sum of BBS task scores: high risk (between 0–20), medium risk (21–40), and low risk of fall (41–56) (see Section 1.1). The risk of fall category, calculated from the physiotherapist scores on the subjects, served as the labels of the training data. An SVM classifier [78] was trained for predicting the fall risk category. The Radial Basis Function (RBF) [79] was used as the SVM kernel, with $\gamma =1/n_{f}$, where $n_{f}$ is the number of features, and the regularization parameter C=3. Leave-one-out cross-validation [74] was used to evaluate the model’s performance.

### 4.4. Automatic BBS Score Prediction Results

We tested the performance of the random forest models in predicting each of the BBS task scores and the SVM classifier in predicting the final fall risk category from the 14 task scores.

Table 1 shows the accuracy of the random forest score predictors for each of the 14 BBS tasks. BBS task scores are in 0–4. The number of samples (*N*) differed between tasks due to some patients’ inability to perform tasks or due to technical difficulties in recording (such as occlusion of the subject by the physiotherapist when protecting the patient from falling). Additionally, the distribution of samples across the possible scores was not even since some tasks were very easy (e.g., sitting in a chair in Task 3) and always scored high grades. As seen in the table, the Mean-Squared Error (MSE) of the classifications was very low across tasks, implying that when the classification was incorrect, it was at most one score unit in error. In addition, we also calculated the weighted precision, recall, and F1-score.

It can be seen that the accuracy varied across the different BBS tasks with some tasks showing low performance. However, considering the end goal of assessing the final fall risk, we show that the predicting model compensated for these inaccurate task score predictions and correctly assessed the final risk with high accuracy.

**Figure 5:**
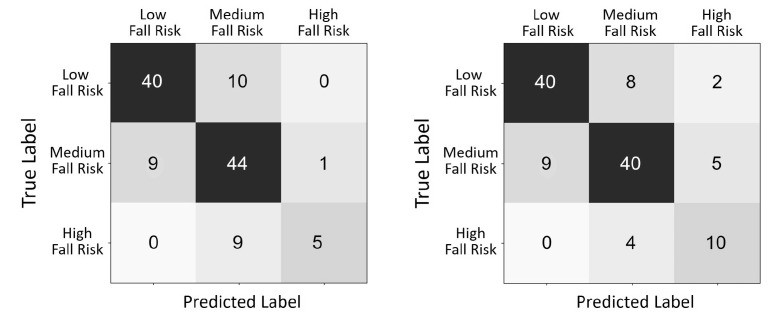
Confusion matrix between the true risk of fall as determined by the physiotherapists and the predicted risk of fall (**left**). False negatives can be reduced by manipulating the thresholds (**right**). The MSE values are 0.25 and 0.29, respectively.

Figure 5a shows the accuracy results in predicting the final risk of fall in one of three categories (high, medium, and low risk of fall). Results are shown as a 3 × 3 confusion matrix comparing the true risk of fall class as determined by the physiotherapists (the sum of the BBS scores assigned by the physiotherapists) with the predicted risk of fall. The overall accuracy was 75.5% correct with an MSE of 0.25. A concern in assessing the risk of fall is the false negative rate (e.g., nine subjects at high risk were classified as medium risk). The ML algorithm allows reducing the false negative rate by adjusting the thresholds. Figure 5b shows the confusion matrix obtained when reducing the false negatives to four subjects. This, however, incurred an increase in false positives and in the MSE (to 0.29).

Finally, feature ranking was performed on the final fall risk prediction model. Features were ranked according to their F-statistic [80]. The most predictive features were found to be:

- Turn 360° (Task #11);
- Alternate feet on step (Task #12);
- Transfers between chairs (Task #5);
- Reaching forward with outstretched arm (Task #8).

Indeed, the first two are considered in practice to be highly informative (as confirmed by the physiotherapists who co-authored this paper).

### 4.5. Statistical Analysis

Statistical analysis was performed to evaluate the correlations between the physiotherapist scores of the BBS and the predicted scores produced by our automated system (termed ML predictions). Two physiotherapists scored each of the patients performing the 14 BBS tasks. For each patient, an ML prediction was calculated for each BBS task. The overall level of risk was categorized into three risk levels: high, medium, and low risk of fall. The overall level of the risk of fall was determined by the sum of the 14 scores: 0–20: high fall risk; 21–40: medium fall risk; 41–56: low fall risk.

An intraclass correlation (two-way mixed-model, single measure) [81] was used for measuring inter-rater reliability of the BBS final score between the two physiotherapists and the ML prediction. Included in the analysis also was the minimal score between the two physiotherapists (MIN(A,D)), calculated on each sample independently. This is in accord with a conservative scoring that tends toward fewer false alarms (see Section 4.4). AN Intraclass Correlation Coefficient (ICC) above 0.8 reflects high reliability, 0.6–0.79 moderate reliability, and less than 0.6 low reliability. Table 2 shows the ICC results. The ICC measure of the raters’ consistency in measuring final BBS scores was higher between the physiotherapists than between the physiotherapists and the ML prediction (Table 2). Saying that, the correlation between the prediction results and the physiotherapists’ measures was high (>0.83) both between the two physiotherapists and between each physiotherapist and the ML prediction.

## 5. Efficient Fall Risk Evaluation Algorithm

The automated system for BBS assessment presented above is an effective method for reducing physiotherapist resources and allowing a wider screening of the elderly community for the risk of fall. In this section, we introduce an additional enhancement in which machine learning was used to reduce the number of BBS tasks required to be performed. This approach can reduce the number of tasks from 14 to an average of 4–6 tasks per subject, thus reducing the amount of time spent by the patient and the medical staff member (physiotherapist or the person supervising the automatic process) required for assessing fall risk. The approach can be applied both to the physical BBS and to the automatic system and in essence can be exploited for any other battery of tests.

The standard BBS assessment carried out either by a physiotherapist or performed using the automated method described above includes 14 BBS tasks that are performed by the subject in a predefined sequential order. The subject is scored on each of the tasks. The scores are then either summed (if collected by the physiotherapist) or run through our automated ML algorithm (Section 4) in order to assess the final fall risk of the subject into one of three classes (high, medium, or low fall risk).

Considering the BBS assessment as an iterative process (where one task is performed per iteration), every iteration can be considered as a “partial predictor” of the final fall risk assessment category. As additional tests are performed and task scores are accumulated, the prediction becomes more accurate. Thus, we used ML to develop a method in which the BBS tasks were ordered in a manner that optimized for accuracy of the final fall risk prediction and allowed for the testing to terminate early when the prediction reached a high confidence level. The BBS tasks may be administered in a predetermined optimal order constant across all subjects or may be adaptively determined per subject. Either way, the number (and consequently, the time required to perform the BBS assessment) was significantly reduced, making the whole process more efficient.

### 5.1. Preprocessing: Building a Dataset of Fall Risk Predictors

The goal of the adaptive fall risk evaluation algorithm was to find the minimal subset of BBS tasks that would ensure the highest classification (prediction) accuracy for the risk of fall. To this end, we built a dataset of ML-based fall risk predictors. We considered all subsets of the 14 BBS tasks ($2^{14}-1$ subsets) and, for each subset, trained a machine learning classifier to predict the final fall risk assessment using as the input only the scores associated with the tasks in the subset. Together with the prediction, each classifier also output a measure of confidence in the prediction.

The fall risk predictors were trained using the patient data collected for the automated BBS system as described in Section 4. We created two different datasets of predictors. One dataset consisted of predictors trained on the physiotherapists’ BBS scores with the ground truth risk category determined by the sum of these scores. The second dataset consisted of predictors trained on the BBS scores computed by our automated BBS assessment system described in Section 4. The fall risk category determined by the physiotherapists served as the ground truth in this case as well. Three types of machine learning algorithms were tested as predictors: SVM [78], decision trees [82] and random forest [72]. Each of these algorithms outputs the predicted risk class, as well as the confidence in the prediction. The random forest models produced the most accurate predictors, both in terms of accuracy and in terms of the average confidence level. Thus, we considered only the random forest models in this study. The random forests were trained with 100 trees.

For each dataset, the trained predictors were ranked according to the accuracy in prediction (proportion of correct fall risk predictions), as well as the average confidence of the predictions over the training set.

### 5.2. Efficient Re-Ordering of the BBS Tasks

**Figure 6:**
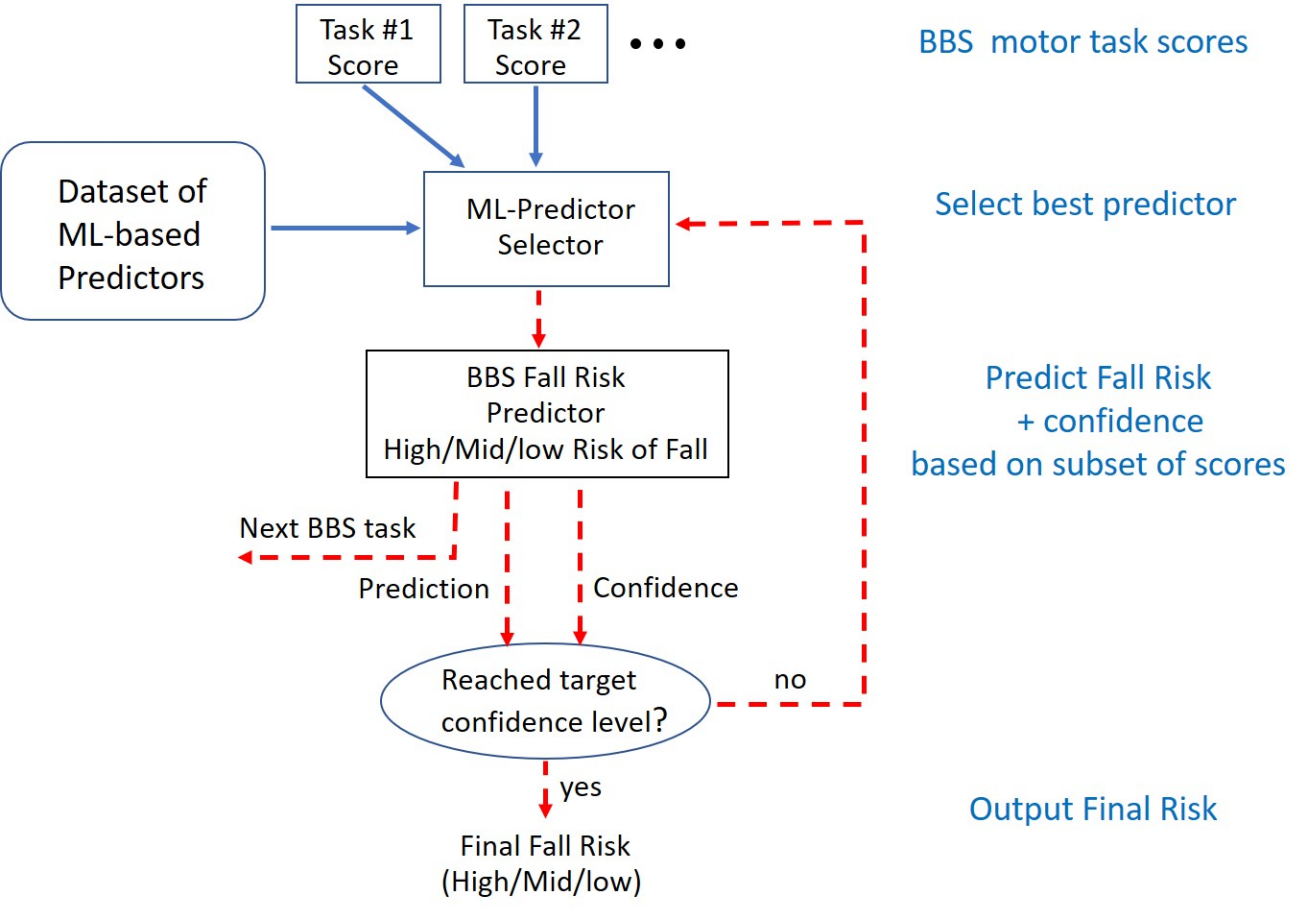
Schematic diagram of the E-BBS fall risk prediction system with efficient and adaptive ordering of the BBS tasks.

The enhancement of the BBS testing that we propose involved re-ordering the BBS tasks and interactively predicting the risk of fall after each task is performed and scored. Together with the fall risk prediction, the confidence in the prediction is given after each task as well. Given a confidence threshold CT, the BBS testing terminates when the confidence exceeds the threshold. A schematic diagram of the system is shown in Figure 6. We term the new ordering and shortened sequence of BBS tasks Efficient-BBS (E-BBS), where the process is efficient in the number of tasks the patient has to perform.

The algorithm for determining the E-BBS task order requires: (a) the first BBS task (or a subset of initial tasks) and (b) a method to determine the next BBS task to perform. Let $x_{i}$ be the BBS scores of the *i*th subject in the training set and be $y_{i}$ the risk class (high, medium, low) associated with $x_{i}$ (assume there are *N* such pairs ($x_{i},y_{i}$)). Recall that the preprocessing step (Section 5.1) created a dataset of ML-based predictors for every subset of the BBS tasks. We define $Pred(SS,x_{i})$ as the fall risk class prediction for $x_{i}$ according to the trained predictor associated with the BBS task subset SS. The function $Conf(SS,x_{i})$ returns the confidence associated with the prediction. E-BBS is an iterative process with a single BBS task performed at each iteration. Let CSS be the current subset of BBS tasks (tasks that have been performed and scored), and denote by NT the next task to be determined from among the unused set of tasks UT.

We developed and tested four different selector methods (see Figure 6) for choosing the next BBS task to be performed:

- Method 1. The next task NT is selected as that which when augmented to CSS creates a subset whose predictor has the highest accuracy over the complete training set.
  $$NT=\arg\max_{T\in UT}\sum_{i=1}^{N}I\left(Pred\left(\left\{CSS,T\right\},x_{i}\right)=y_{i}\right),$$
  where I is the indicator function;
- Method 2. NT is determined as above, but with the accuracy score of the augmented subset predictor calculated only on the training examples $x_{i}$ for which the CSS predictor gives a confidence below the confidence threshold CT, i.e., the $x_{i}$’s for which the classifier did not yet make a decision.
  $$\begin{array}{ccc} NT & = & \arg\max_{T\in UT}\sum_{i=1}^{N}I\left(Pred\left(\left\{CSS,T\right\},x_{i}\right)=y_{i}\right)\\ & & \times \;I(Conf\left(CSS,x_{i}\right)<CT); \end{array}$$
- Method 3. The third method is an adaptive method that depends on the scores $x_{p}$ of the patient being tested for BBS. NT is determined as above, but the *i*th training example’s contribution to the sum is weighted by its similarity to the scores $x_{p}$ of the patient. The greater the similarity, the higher the weight is.
  $$\begin{array}{ccc} NT & = & \arg\max_{T\in UT}\sum_{i=1}^{N}I\left(Pred\left(\left\{CSS,T\right\},x_{i}\right)=y_{i}\right)\\ & & \times \;I(Conf\left(CSS,x_{i}\right)<CT)\\ & & \times \;d(CSS\left(x_{i}\right),CSS\left(x_{p}\right)), \end{array}$$
  where $CSS(x_{p})$ and $CSS(x_{i})$ are the BBS scores of the patient and of the *i*th training sample restricted to the tasks in CSS. As a similarity measure, we used $d\left(x_{i},x_{j}\right)=\exp(-||x_{i}-x_{j}{\left|\right|}^{2}/\sigma^{2})$, where the parameter $\sigma^{2}$ controls the contribution of the point as a function of the distance;
- Method 4. The fourth method extends the third method by considering only the examples in the training set for which the algorithm correctly classified the example.
  $$\begin{array}{ccc} NT & = & \arg\max_{T\in UT}\sum_{i=1}^{N}I\left(Pred\left(\left\{CSS,T\right\},x_{i}\right)=y_{i}\right)\\ & & \times \;I(Conf\left(CSS,x_{i}\right)<CT)\\ & & \times \;d(CSS\left(x_{i}\right),CSS\left(x_{p}\right))\\ & & \times \;I(y_{i}={\hat{y}}_{i}), \end{array}$$
  where ${\hat{y}}_{i}$ is the final prediction of the algorithm, i.e., $Pred\left(AT,x_{i}\right)={\hat{y}}_{i}$, where AT is the set of all tasks.

It can be seen that the first two selector methods produced a task sequence that was independent of the patient input. Thus, these selector methods produced a constant order of BBS tasks that was later used on all patient data when testing. Selector Methods 3 and 4 are adaptive, as the NT task is chosen based on training data, which are dependent on the data of the patient being tested. Thus, for each patient, a different BBS sequence of tasks is produced. However, we show later in Section 5.3 that, in fact, all E-BBS sequences shared the same initial portion of the task sequence.

### 5.3. Results: Efficient BBS

Given a starting subset of BBS tasks, a confidence threshold, and a training set, each of the four selector methods produces a different optimal ordering of BBS tasks. To evaluate the performance of each such ordering, we used five-fold cross-validation on the training set. For consistency, we also compared the results with the standard ordering of BBS tasks [7,8], as well as the Short-Form BBS (SFBBS), which selected a subset of seven tasks to be performed [34] (see Section 1.1).

The quality of the performance of a specific ordering of tasks was evaluated using two measures: the accuracy of predicting the fall risk category and the average number of BBS tasks required to complete the prediction process. Since the Efficient-BBS assessment terminates the testing when the confidence of the prediction reaches the desired threshold, the number of required BBS tasks was significantly lower than the number of BBS tasks in the standard BBS test (14).

We compared the performance of the adaptive ordering across selector methods, using confidence thresholds of 90, 92, 94, 96, 98, and 100. The initial subset of BBS tasks considered were of size 1, 2, and 3 (a discussion on the significance of starting with an initial subset of tasks is given in the Discussion Section 6). Finally, we compared the results across the two types of datasets: based on the physiotherapist scoring and based on the automatic BBS scoring.

**Figure 7:**
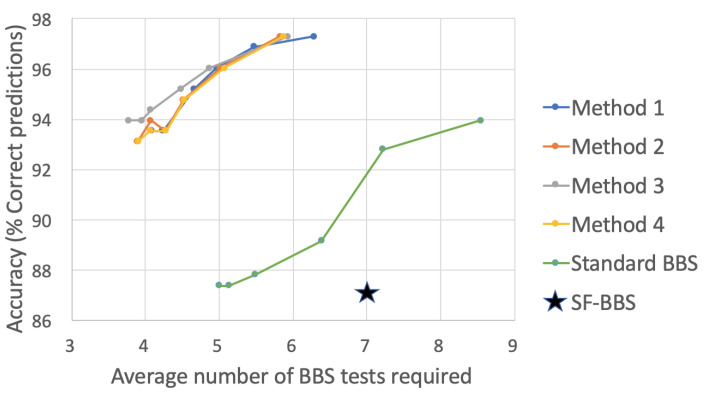
Accuracy vs. average number of BBS tests for different selector methods (Section 5.2) trained on the physiotherapist scoring. For each method, the plot shows values for 6 different confidence thresholds (90, 92, 94, 96, 98, and 100).

Figure 7 plots the accuracy and the average number of BBS tasks required for the E-BBS ordering produced by the four selector methods trained on the physiotherapists scoring, as well as the standard BBS ordering. For each method, the plot shows values for the six different confidence thresholds. Naturally, the higher the confidence threshold, the longer the length of the sequence is. The initial test set was selected as the optimal set of three tasks, as discussed below, and included the three BBS tasks numbered {8,9,11} (see [7]). As can be seen, all orderings of BBS tasks reached an accuracy of around 97% correct risk of fall predictions. However, the different selector methods showed a significant reduction in average BBS tasks compared to the standard BBS, requiring from 4–6 tasks on average compared to the 14 tasks of the standard BBS. Additionally, we plot the performance of the SF-BBS [34] with seven BBS tasks at an accuracy rate of 87% on our patient data, showing that the E-BBS significantly outperforms the SF-BBS (the SF-BBS uses a three-unit scoring scale, whereas we relied on a five-unit scale used in the standard BBS testing). The four selector methods showed comparable performance with a slight advantage for Method 3.

**Figure 8:**
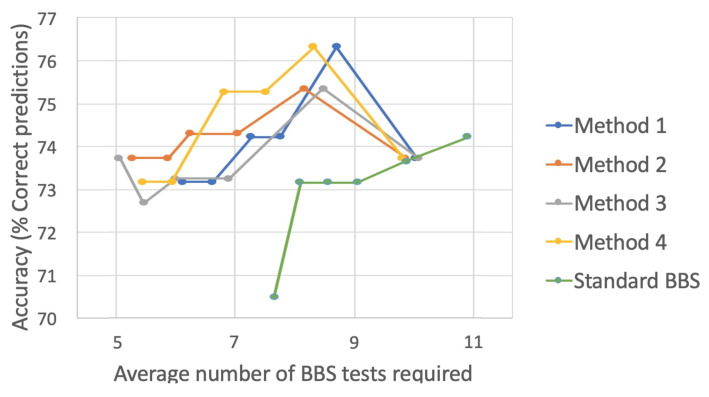
Accuracy vs. average number of BBS tests for different selector methods (Section 5.2) trained on the automatic BBS scoring. For each method, the plot shows values for 6 different confidence thresholds (90, 92, 94, 96, 98, and 100).

Figure 8 displays the same results as Figure 7 when training was performed on the scores predicted by the automatic BBS system. One can observe a lower rate of performance, but, as before, the standard BBS was strongly outperformed by the four selector methods, with Method 4 showing the best performance. However, in this case, all methods reached an accuracy of 76–77% correct risk of fall classification. Furthermore, it can be observed that there was a drop in accuracy when the confidence threshold reached 100. This was due to the fact that the automatic BBS score assessment was inconsistent in its performance with some of the BBS tasks showing low prediction accuracy, as shown in Table 1. The trained predictors selected the high-accuracy tasks first in the E-BBS ordering, leaving those with low accuracy to later in the ordering. When the confidence threshold was low, the BBS assessment of a subject was able to predict confidently without relying on those BBS tasks with low accuracy. However, when the confidence threshold approached 100, those tasks must be recruited, and their inaccuracy led to incorrect predictions of the overall fall risk. Albeit that there was this fault, the average number of required BBS tasks was still significantly lower than 14. We note that when continuing up to the fourteenth task, the four selector methods did not improve in accuracy beyond that shown in the plot, which is consistent with the non-adaptive results shown in Figure 5.

We now question the initial BBS tasks used by the E-BBS test. The reason for allowing a definition of an initial set of BBS tasks is that the iterative method of BBS testing and the design of the selector methods inherently imply that the optimal ordering was determined following a greedy algorithm. As such, a local minimum may be reached in the optimization. To mitigate this effect, we allowed a global optimal subset to be chosen as the initial set of tasks in the ordering.

Without any external constraints on the initial task set, we chose the set to be that which performed optimally. Since the predictors trained in the preprocessing stage (Section 5.1) were each ranked by their prediction accuracy, we chose a subset of a predefined size whose predictor showed the best accuracy. We considered subsets of size 1, 2, and 3. Table 3 shows the accuracy of the predictors associated with subsets of size 1 when trained on the patient data with physiotherapists’ scoring. The results in the table can be interpreted as the predictive quality of each individual task of the BBS. It can be seen that Task #9, as a single task, was the best predictor of fall risk on our test set with 85.5% accuracy. Similarly, for subsets of size 2 and 3, we found that the optimal initial task sets were {9,11} and {8,9,11}, respectively.

**Figure 9:**
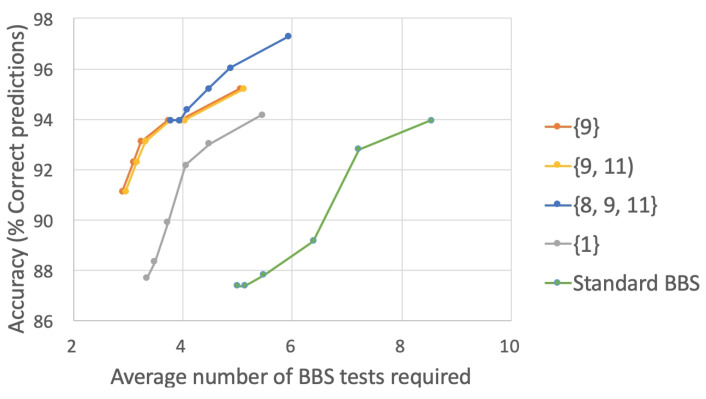
Accuracy vs. average number of BBS tests for the different initial subset of tasks. Results are shown for Selector Method 3 and training on the physiotherapists’ data. For each initial subset of the tasks, the plot shows values for 6 different confidence thresholds (90, 92, 94, 96, 98, and 100).

Figure 9 shows the accuracy vs. the average number of BBS tasks required, when using different initial subsets of BBS tasks. For comparison, also shown are the results for Subset {1} and for the standard BBS test sequence. Results are shown for Selector Method 3. As can be seen, all E-BBS orderings were significantly better than the standard BBS and also better than the Subset {1} case. The accuracy was highest for the subset of size 3, reaching 97% accuracy at a confidence level of 100. All orderings required only 3–6 BBS tasks on average. Using BBS Task 1 as the initial task, as is used in the standard BBS test, showed the least accurate results of the E-BBS orderings. This is indicative of the structure of the standard BBS test where “easier” tasks are performed at the beginning of the testing sequence. These, however, are less informative and have a lower predictive quality (see Table 3). In the optimal ordering, these would appear later in the ordering, with the more informative tasks appearing first.

**Figure 10:**
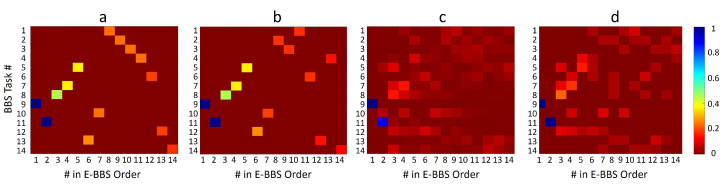
Occurrence matrices depicting the ordering of BBS tasks in the E-BBS. Columns indicate the order in the E-BBS sequence. Each row indicates a standard BBS task as defined in [7]. The matrix entry value indicates the proportion of times a BBS task was used in a certain E-BBS sequence position across the test set. (**a**–**d**) Occurrence matrices of E-BBS sequences as trained on the physiotherapist data and using the 4 Selector Methods 1 to 4, respectively.

**Figure 11:**
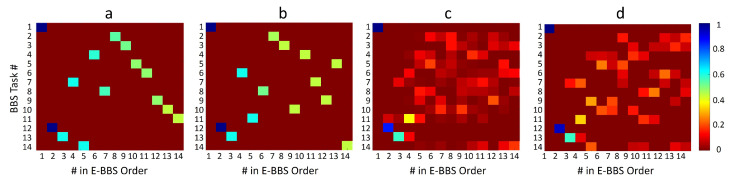
(**a**–**d**) same as Figure 10, but trained on the automatic BBS scoring data.

Finally, we studied the new order of BBS tasks as expressed in the E-BBS. We first considered the physiotherapist training set and, for simplicity, focused on the initial task subset with the single BBS Task #9, which was determined as the optimal starting task, and we set the confidence threshold to 100. We considered the four task selector methods (Section 5.2) and considered the E-BBS task sequence they produced over a test set of patients. To present the results, we used occurrence matrices, as shown in Figure 10 and Figure 11. Columns of the matrix indicate the order in the E-BBS sequence. Each row indicates a standard BBS task enumerated 1–14. The value in each matrix entry (i,j) indicates the proportion of times that BBS task i appeared in an E-BBS sequence in position j across all E-BBS sequences produced over the test set.

Figure 10 displays four occurrence matrices trained and tested on the physiotherapist data. Matrices (a) to (d) show results for Selector Methods 1 to 4, respectively. It can be seen that the number of BBS tasks used in the E-BBS sequences decreased along the order. This was due to the fact that for most patients, the number of tasks required to reach the confidence threshold was much lower than 14, and the E-BBS evaluation was terminated before all 14 tasks were performed.

As expected, Selector Methods 1 and 2, which are not-adaptive, produced a constant sequence of the E-BBS, which is a permutation of the standard BBS. Selector Methods 3 and 4 are adaptive and thus produced a different E-BBS sequence for each subject. However, it can be seen that the first two tasks in the sequence were always the same—Tasks #9 and #11 (followed by #8 with high probability)—and then showed variability in the subsequent tasks, with Selector Method 3 showing a wider variability than Method 4. More interesting is the fact that the initial part of the E-BBS sequence was similar across all four selector methods (all four matrices showed initial BBS Tasks 9, 11, 8, and even 7 with high values). This indicates that regardless of whether the adaptive or constant E-BBS is used, the same BBS tasks will be invoked initially, implying that these tasks are predictive of the final assessment of the risk of fall.

Figure 11 displays similar occurrence matrices trained on the automatic scoring of BBS patients. Here too we see similar characteristics, albeit noisier. The common initial tasks in the E-BBS sequence on these data were BBS Tasks 1, 12, and 13. The distinction between this sequence and that obtained for the physiotherapist data was due to the fact that the automatic system introduces errors in the BBS scoring itself. Thus, the tasks appearing early in the E-BBS are those that are predictive of fall risk, as well as reliable in terms of automatic BBS scoring.

The outcome of this analysis implies that the E-BBS order of BBS tasks can be set as constant for the first three tasks (namely, Tasks 9, 11, and 8), followed by either the constant sequence determined by Selector Methods 1 and 2 or performed adaptively per patient using Selector Methods 3 or 4. Considering that most patient testing terminated early due to reaching the desired confidence level, the E-BBS sequence beyond the first 3-6 tasks was rare.

We summarize the orderings of tasks in the E-BBS testing in Table 4 and Table 5. In Table 4, sequences are shown for Selector Methods 1 and 2 and for the physiotherapist data and the automatic scoring data. As described above, the first three tasks are common to all E-BBS options, diverging only later. Regarding Methods 3 or 4, tasks were selected adaptively for each subject according to the BBS scores achieved until this step. Table 5 shows an example of a single subject for both methods. Note that this task sequence terminated at different points for each subject dependent on the subject’s scores and the configured confidence threshold CT.

## 6. Discussion and Conclusions

We presented an approach to automating the BBS fall risk assessment test. The approach involves two main parts. First, a computer vision and ML-based system tracks the motion and pose of human subjects performing the BBS tasks, and then, a two-level ML model first predicts the BBS score for each of the fourteen tasks, the output of which is fed into another ML model, which then predicts the final fall risk category. In addition, we presented an ML-based method that determines an Efficient-BBS (E-BBS) battery of tests, requiring the patient to perform only a subset of the original BBS tests, while achieving the same quality of prediction as the full BBS test in a significantly shorter time. We emphasize that the E-BBS can be implemented on the outputs predicted by the automated BBS score predictor or directly on the scores supplied by the physiotherapists.

The approaches presented in this paper were tested on data collected at a major hospital where physiotherapists provided BBS scores and the level of fall risk for hospital patients and healthy subjects. The system showed high accuracy rates on assessing fall risk and good correlation with ground truth scores on the individual BBS tasks. In our experiments, we used real test results, where the tests were performed in the standard order, but we simulated the order of the tests for the E-BBS evaluation. In a real setting, the physiotherapists (our co-authors) stated that the order of tests has some importance and starting first with easier tests might produce better scores by the patients. Thus, additional considerations could be added into the subset selection process, possibly incurring a slight decrease in performance. This is a topic of future research.

The complete system is non-invasive and easy to use in a set-up-and-go form, well suited to be used by non-technically-savvy individuals. Furthermore, the E-BBS allows the testing to be significantly more time efficient. Thus, the system is well suited for expanding testing beyond the confines of hospitals, medical centers, and doctors’ offices. It allows implementing a wide-scale screening of the elderly population for a high risk of fall. The system can efficiently determine those at low risk and, more importantly, direct those found to be at high risk to further medical assessment and preventive treatment.

Finally, we note that this study focused on evaluating the risk of fall and the BBS scores. However, the motion analysis, as well as the efficient sequencing approach can be applied to any other sequence of assessment tests.

## Figures and Tables

**Table 1:** Automatic prediction of BBS scores per task.

BBS Task	Task Description	N	Samples per Class <0,1,2,3,4>	Accuracy	MSE	Recall	Precision	F1
1	Sitting to Standing	102	0,0,0,66,36	87%	0.18	0.87	0.88	0.87
2	Standing Unsupported	111	0,0,15,24,72	73%	0.36	0.73	0.71	0.71
3	Sitting with Back Unsupported	112	0,0,0,0,0,112	100%	0.0	1	1	1
4	Standing to Sitting	105	0,0,0,53,52	88%	0.15	0.88	0.88	0.88
5	Transfers	96	0,0,22,39,35	72%	0.36	0.72	0.72	0.72
6	Standing Unsupported, Eyes Closed	101	0,0,0,49,52	71%	0.32	0.71	0.72	0.71
7	Standing Unsupported, Feet Together	106	13,13,0,33,47	72%	0.37	0.72	0.72	0.72
8	Reaching Forward	75	0,17,0,24,34	73%	0.51	0.73	0.72	0.72
9	Pick up Object from the Floor	99	7,0,0,39,53	72%	0.31	0.72	0.74	0.70
10	Look Behind Shoulders	102	7,9,8,32,46	52%	1.25	0.52	0.50	0.51
11	Turn 360°	100	14,26,20,7,33	66%	0.60	0.66	0.62	0.64
12	Alternate Feet on Step	93	39,11,12,0,31	74%	0.34	0.74	0.69	0.71
13	Standing Unsupported, One Foot in Front	93	30,14,30,0,19	68%	0.54	0.68	0.64	0.64
14	Standing on One Leg	109	39,40,8,0,22	66%	0.80	0.66	0.64	0.65

**Table 2:** The intra-class correlation coefficient between physicians and ML of the BBS scores. All *p*-values < 0.001.

	D	Min(A,D)	ML Prediction
A	0.981	0.989	0.839
D		0.992	0.834
Min(A,D)			0.824

**Table 3:** Singe BBS tasks—predictor accuracy.

BBS Task	Accuracy (%)
9	85.5
7	81.4
6	81.2
11	80.8
8	80.0
4	77.8
5	77.4
12	76.2
1	74.2
10	72.6
2	70.7
13	67.5
14	67.3
3	50.8

**Table 4:** E-BBS order of tasks using Methods 1 and 2. Task numbers are the standard BBS task numbers [7].

Data	Method	T	T	T	T	T	T	T	T	T	T	T	T	T	T
	#	1	2	3	4	5	6	7	8	9	10	11	12	13	14
Physiotherapist	1	9	11	8	7	5	13	10	1	2	3	4	6	12	14
	2	9	11	8	7	5	12	10	2	3	1	6	13	4	14
Automatic	1	1	12	13	7	14	4	8	2	3	5	6	9	10	11
	2	1	12	13	6	11	8	2	3	10	4	7	9	5	14

**Table 5:** E-BBS order of tasks using Methods 3 and 4. Task numbers are the standard BBS task numbers [7].

Data	Method	T	T	T	T	T	T	T	T	T	T	T	T	T	T
	#	1	2	3	4	5	6	7	8	9	10	11	12	13	14
Physiotherapist	3	9	11	8	7	4	5	10	1	3	14	2	13	12	6
	4	9	11	8	7	2	5	10	1	4	3	6	13	14	12
Automatic	3	1	12	13	11	5	4	9	2	10	14	8	7	6	3
	4	1	12	13	11	4	5	7	10	14	8	2	9	3	6
